# The Angiotensin II Type 2 Receptor in Brain Functions: An Update

**DOI:** 10.1155/2012/351758

**Published:** 2012-12-25

**Authors:** Marie-Odile Guimond, Nicole Gallo-Payet

**Affiliations:** Division of Endocrinology, Department of Medicine, Faculté de Médecine et des Sciences de la Santé, Université de Sherbrooke, Sherbrooke, QC, Canada J1H 5N4

## Abstract

Angiotensin II (Ang II) is the main active product of the renin-angiotensin system (RAS), mediating its action via two major receptors, namely, the Ang II type 1 (AT_1_) receptor and the type 2 (AT_2_) receptor. Recent results also implicate several other members of the renin-angiotensin system in various aspects of brain functions. The first aim of this paper is to summarize the current state of knowledge regarding the properties and signaling of the AT_2_ receptor, its expression in the brain, and its well-established effects. Secondly, we will highlight the potential role of the AT_2_ receptor in cognitive function, neurological disorders and in the regulation of appetite and the possible link with development of metabolic disorders. The potential utility of novel nonpeptide selective AT_2_ receptor ligands in clarifying potential roles of this receptor in physiology will also be discussed. If confirmed, these new pharmacological tools should help to improve impaired cognitive performance, not only through its action on brain microcirculation and inflammation, but also through more specific effects on neurons. However, the overall physiological relevance of the AT_2_ receptor in the brain must also consider the Ang IV/AT_4_ receptor.

## 1. Introduction

A major advance in the field of the renin-angiotensin system (RAS) was the discovery of a complete RAS in the brain, independent from the peripheral system, by Jacques Genest's laboratory in Montreal in 1971 [1] (for reviews see [2–4]). Further studies by Mendelsohn et al. [5, 6] and Unger et al. [7] corroborated these observations, using biochemical, pharmacological, and autoradiographic approaches. It was found that brain levels of angiotensin II (Ang II) are higher than its circulating levels, suggesting independence of the two systems (review in [3, 4]). The various components of RAS (angiotensin-converting enzyme (ACE), Ang II and Ang II receptors) are all found in the adult brain in areas involved in the regulation of fluid and electrolyte balance, in the regulation of arterial pressure and vasopressin release, and regulation of the autonomic system [8, 9]. They are also present in structures involved in cognition, behavior, and locomotion. In particular, over the past 10 years, several advances have been made regarding the role of Ang II in various brain functions, including cerebroprotection, stress, depression, and memory consolidation [3, 4, 10–12]. Moreover, many evidences highlight the potential function of Ang II in the etiology of certain neurodegenerative diseases, including Alzheimer's and Parkinson's disease, seizures, and the development of metabolic syndrome and diabetes (review in [4, 13, 14]). 

**Figure 1:**
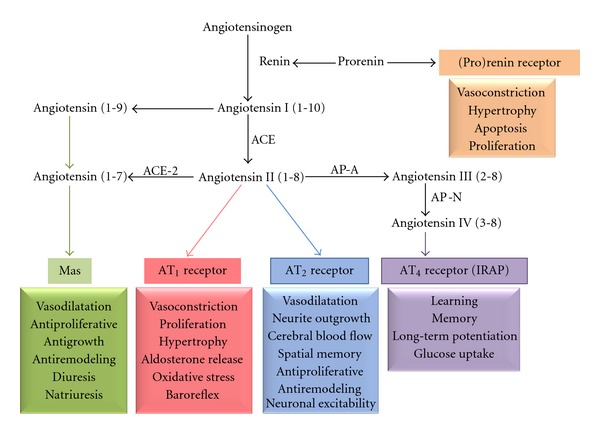
Summary of the brain renin angiotensin system (RAS). The figure summarizes the conversion of angiotensinogen to angiotensins I and II through fragments. The biologically active forms include angiotensins II, III, IV, and (1–7). The main enzymatic pathways are mediated by renin and angiotensin converting enzyme ACE or ACE2, AP-N and AP-A. The major brain effects of angiotensins are mediated by AT_1_, AT_2_, AT_4_, prorenin, and Mas receptors. The functions associated with each receptor are indicated. ACE: angiotensin converting enzyme; AP-A: aminopeptidase A; AP-N: aminopeptidase N, adapted from Phillips and Oliveira, 2008 [3] and from Wright and Harding, 2012 [4].

In the classical view, synthesis of Ang II begins with the conversion of angiotensinogen into Ang I by the enzyme renin. Ang I is then converted by ACE into Ang II (a.a. 1–8), which is then metabolized to Ang III (a.a. 2–8) and Ang IV (a.a. 3–8). Ang II and Ang IV can be further converted into Ang (1–7) and Ang (3–7) (Figure 1). Ang II binds to two main receptors, namely, the angiotensin type 1 (AT_1_) and type 2 (AT_2_) receptors, both belonging to the G-protein coupled receptor (GPCR) family [4, 12, 13]. Aside from this common link, these two receptors otherwise carry very little similarities. Indeed, their actions are generally opposite and while the AT_1_ receptor is expressed abundantly in several tissues, expression of AT_2_ receptor is limited to specific tissues and brain areas, where its concentration is generally low compared to the AT_1_ receptor. Most of the known effects of Ang II are due to activation of the AT_1_ receptor, including vasoconstriction, cellular growth, and proliferation. On the other hand, it is generally assumed that the AT_2_ receptor counteracts the action of the AT_1_ receptor, promoting vasodilatation, apoptosis, and antigrowth effects. In addition to the classical AT_1_ and AT_2_ receptors, more recent studies have identified other receptors for RAS components, such as the (pro)renin receptor, the Ang (1–7) Mas receptor, and the Ang IV receptor (AT_4_ receptor, called IRAP for insulin-regulated aminopeptidase), all of which are expressed in the brain (for reviews, see [4, 12, 13]) (Figure 1). In particular, several studies have shown that Ang IV-AT_4_ receptor/IRAP have important functions in the brain, related to cognition and memory. In addition, in many situations, Ang IV acts as an inhibitor of AT_1_ receptor actions [15–17]. 

The present paper is focused on the known and suggested roles of the AT_2_ receptor in brain functions related to neuronal activities and cognitive disorders as well as the potential link between metabolic syndrome and cognitive functions. The role of the AT_2_ receptor in the central regulation of blood pressure, thirst and related pathologies, such as hypertension, stroke, or ischemic damage, will not be discussed here; these latter topics are covered in recent reviews (see [11, 18–20]). For further detail, readers are also invited to consult several excellent reviews describing recent up-to-date advances pertaining to the various active Ang II-derived ligands and their receptors [4, 12, 13] However, considering that there are some similarities between the AT_2_ receptor and the Ang IV/AT_4_ receptor, comparisons between the two will be included, when appropriate.

## 2. The Type 2 Receptor—A Nonclassical GPCR

Three important periods highlight the history of the AT_2_ receptor: (i) its discovery in the 1980s, (ii) its cloning in 1991–1993, and (iii) the generation of transgenic mice in 1995. However, because Ang II has a similar affinity for both of its receptors, it has been difficult to discriminate between the latter using nonselective ligands such as native Ang II or peptide analogs such as Saralasin or SarIle. At the end of 1980s, tools enabling to distinguish these Ang II receptors became available. The first tools included the nonpeptide antagonists losartan (previously known as DUP753) and PD123,177, and peptide ligands such as CGP42112A and p-aminophenylalanine (review in [2]). Seminal autoradiographic studies [5, 6], followed by biochemical studies by the groups of Marc de Gasparo, Peter Timmermans, and Robert Speth revealed the presence of two binding sites for Ang II, which differed in their expression pattern and biochemical properties [21–25]. However, the conclusive evidence for the existence of two receptors came from their cloning in the early 1990s [26, 27]. Despite the fact that both receptors belong to the large 7-transmembrane domain family of GPCR, AT_1_ and AT_2_ share only ~34% amino acid sequence identity. Moreover, while the AT_2_ receptor displays most of the structural features of a GPCR, it is usually considered as an atypical member of this family. Indeed, although some of its signaling pathways involve a G_i_-dependent mechanism, most of the known effects of the AT_2_ receptor are independent from G-protein coupling mechanisms (for reviews, see [14, 20, 28–31]). Moreover it fails to induce all of the classical signaling pathways such as cAMP, production of inositol triphosphate, or intracellular calcium release (for reviews, see [28, 29]). 

### 2.1. Selective Ligands of the AT_2_ Receptor

In fact, AT_2_ receptor functions are still unclear both in many physiological and pathophysiological situations, mainly because research on this receptor has long been hampered by at least three challenges, namely, (i) the low and unusual expression of this receptor, (ii) its nonclassical signaling, and (iii) the absence of appropriate selective ligands. While several nonpeptide ligands with a large spectrum of selectivity for the AT_1_ receptor are readily available, only a few have been developed for the AT_2_ receptor. Until recently, physiological and pharmacological assessments of the AT_2_ receptors were obtained using either the antagonist PD123,319 (a modification of the initial PD123,177) or the more common AT_2_ receptor agonist CGP42112A, although two other peptides have also shown agonistic properties on AT_2_ receptors, namely, the agonist *p*-aminophenylalanine [22] and novokin [32, 33]. However, the fact that CGP42112A is only a partial agonist, combined with its short half-life, has hampered its further use in *in vivo* studies (review in [20, 34, 35]). Thus, many hypotheses regarding AT_2_ receptor functions in physiological situations have emerged from indirect observations, either using transgenic animals, or blockade of the AT_1_ receptor. In 2004, Wan and collaborators synthesized the first selective nonpeptide AT_2_ receptor agonist, called compound 21 (C21) [36], now renamed M024 [37]. Since then, an increasing number of studies have used C21/M024 for both *in vitro* and *in vivo* studies (see further below) to better investigate the selective role of the AT_2_ receptor (for review, see [20, 34]).

## 3. Overview of the Signaling Pathways of the AT_2_ Receptor in the Brain 

Signaling pathways associated with the AT_2_ receptor primarily involve a balance between phosphatase and kinase activity. The final outcomes vary according to whether the cell is undifferentiated or differentiated and whether angiotensin AT_1_ receptors are also expressed or not. Although there is still much controversy surrounding the effects of the AT_2_ receptor in peripheral systems, its role and mechanisms of action in the brain have gained much greater consensus [14, 31, 38]. 

### 3.1. Signaling Pathways of the AT_2_ Receptor Leading to Neurite Outgrowth

**Figure 2:**
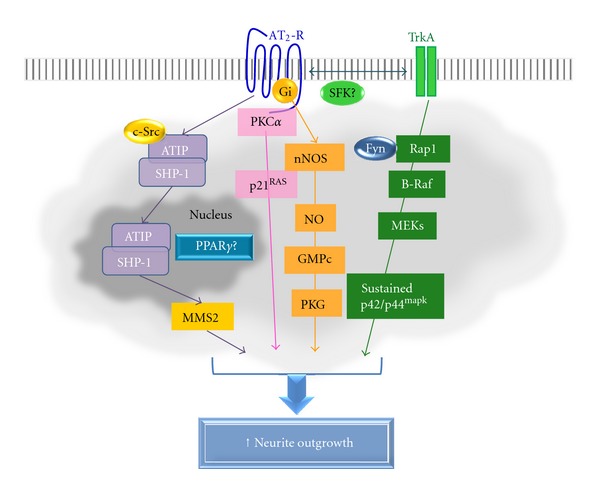
Overview of the main signaling pathways implicated in the action of AT_2_ receptor leading to neurite outgrowth.

In neuronal models, studies on functionality and signaling have usually been conducted simultaneously. Most of the data, including our own findings using NG108-15 cells, indicate that the effects of the AT_2_ receptor on neurite outgrowth involve four main complementary signaling cascades (Figure 2). The first cascade entails a decrease in p21^RAS^ [39] and protein kinase C*α* (PKC*α*) activities [40], both of which are involved in the switch from proliferation to differentiation. Simultaneously, a second cascade, initiated by Rap1/B-Raf, induces a delayed and sustained phosphorylation of p42/p44^mapk^ [39, 41]. In both NG108-15 [39] and PC12W [42] cells, this sustained activation is essential for inducing neurite outgrowth. The initial activation of Rap1 by the AT_2_ receptor is not direct, but rather mediated by phosphorylation of the tropomyosin-related kinase receptor A (TrkA) [43], through the intervention of a Src family kinase member [44]. The third cascade comprises nitric oxide (NO) and cGMP. In NG108-15 cells, we [45] and others [46] have shown that external application of NO is sufficient to induce neurite outgrowth and elongation. In these cells, neuronal NO synthase (nNOS) activation and cGMP production induced by the AT_2_ receptor is dependent on the G*α*_i_ protein. However, cGMP is not involved in Ang II-induced activation of p42/p44^mapk^ [45]. More recently, Li et al. [47] have shown that after AT_2_ receptor stimulation, the AT_2_ receptor interacting protein, ATIP [48], interacts with the tyrosine phosphatase SHP-1. The complex then translocates into the nucleus where it transactivates the ubiquitin-conjugating enzyme variants called methyl methanesulfonate sensitive 2 (MMS2), resulting in neural differentiation and protection (reviewed in [31, 49]). Finally, interaction of AT_2_ with certain receptor tyrosine kinases may also induce neurite outgrowth. For example, in a model of fructose-induced insulin-resistant rats, authors also demonstrated that neurite outgrowth of dorsal root ganglia (DRG) neurons induced by the AT_2_ receptor was facilitated by activation of the phosphatidylinositol 3-kinase (PI3K)/Akt pathway, suggesting the existence of a crosstalk between the AT_2_ receptor and the insulin receptor [50]. In addition, several phosphatases, such as PP2A [51–54] and Src homology region 2 domain-containing phosphatase-1 (SHP-1), are clearly associated with the mechanism of action of the AT_2_ receptor. Finally, certain pathways associated with AT_2_ receptor activation may be through interaction with protein partners such as the promyelocytic zinc finger (PLZF) protein [55, 56] or the peroxisome proliferator-activated receptor gamma (PPAR*γ*) [57] (for review, see [29, 31, 58–60]). 

### 3.2. Ang-II-Independent Effects of the AT_2_ Receptor

It should be noted that while the main effects of AT_1_ and AT_2_ receptors are dependent of Ang II binding, some evidences also suggest that they may have certain ligand-independent effects (review in [29, 31]). For example, AT_1_ receptors can be activated by mechanical stress, independently from Ang II binding and/or stimulation of p42/p44^mapk^ [61, 62]. Similarly, AT_2_ receptor overexpression in CHO cells, R3T3 fibroblasts, and vascular smooth muscle cells enhances apoptosis signaling simply by its overexpression [63]. Another study also observed that the AT_2_ receptor, when expressed as a constitutive homooligomer, leads to G-protein dysfunction and symptoms of neurodegeneration without Ang II stimulation [64]. Although the mechanism underpinning this effect still requires further investigation, it appears that the C-terminal portion of the AT_2_ receptor is essential, since expression of a mutant AT_2_ receptor truncated in its C-terminal region is unable to form oligomers. Aside from its effect on cell survival, Ang II-independent effects of the AT_2_ receptor also include modulation of gene expression, at least in human coronary artery endothelial cells, where AT_2_ receptor overexpression modulates more genes than CGP42112A stimulation [65]. Although these AT_2_-regulated genes are associated with many cellular functions, including cell migration, protein processing, intracellular signaling, and DNA repair, it is still unknown whether Ang II-independent effects of the AT_2_ receptor are associated with protective effects in neuronal function. Moreover, an Ang II-independent effect of the AT_2_ receptor was observed in a model overexpressing the receptor, and thus its relevance in physiological situations is still unknown. Clearly, many questions still remain to be elucidated in order to fully understand how the AT_2_ receptor exerts its effects on brain functions. However, new recent insights in AT_2_ receptor signaling have been achieved which could partly explain some of the observed discrepancies (see Section 8). 

### 3.3. Signaling Pathways Associated with Ang IV and the AT_4_ Receptor

Interestingly, despite the high similarity between central biological effects associated with AT_2_ and AT_4_ receptor stimulation (memory processing, long-term potentiation facilitation, protective function in cognitive loss) (see Section 5), the signaling pathways induced by these two receptors are clearly different. In contrast with the AT_2_ receptor, the AT_4_ receptor is a single transmembrane receptor, initially known for its aminopeptidase activity, leading to peptide processing of oxytocin, vasopressin, Ang III, met-enkephalin, somatostatin, and other neuropeptides (review in [4, 12]). Ang IV has also been shown to increase endothelial NO synthase activity via mobilization of intracellular calcium [66, 67] and to stimulate the PI3 K/PKB (protein kinase B) pathway [68] in endothelial cells. A recent study also showed that Ang IV treatment in diet-induced hyperglycemic mice increased the interaction between IRAP and PI3 K, leading to activation of Akt and the glucose transporter type-4 (GLUT4) translocation. This pathway was also associated with an improvement in glucose tolerance [69], suggesting that Ang IV may be implicated in insulin signaling and development of diabetes. Finally, in some studies, Ang IV was also found to elicit rapid activation of other selected kinases including ERK1/2, p38 kinase, focal adhesion kinase, and paxillin (review in [70]). Nevertheless, it appears that these signaling effects of Ang IV are model dependent, and none of these pathways have been observed in neuronal cells. Therefore, although renewed interest in Ang IV has recently emerged with the recognition that the Ang IV/IRAP/AT_4_ receptor plays an important role in cognition and pain [4, 12, 71], it is still unknown to date whether signaling pathways associated with Ang IV stimulation are implicated in these effects. 

## 4. Expression and Roles of the AT_2_ Receptor in the Brain

All of the various RAS components in the brain, and in particular the AT_2_ receptor, are highly expressed during fetal life, suggesting that they could play key roles during development. On the other hand, in the adult, AT_2_ receptor expression in the brain is limited to certain specific areas (review in [72–75]). For instance, the AT_2_ receptor has been found at high levels in the medulla oblongata (control of autonomous functions), septum and amygdala (associated with anxiety-like behavior), thalamus (sensory perception), and superior colliculus (control of eye movements in response to visual information) as well as in the subthalamic nucleus and cerebellum (areas associated with learning of motor functions). It is also expressed with the AT_1_ receptor in areas involved with cardiovascular functions, learning and behavior (cingulate cortex, molecular layer of the cerebellar cortex, superior colliculus, paraventricular nuclei hippocampus) (for extensive mapping, see [72, 73]). More recently, expression of the AT_2_ receptor was also detected in the substantia nigra pars compacta, the area involved in dopaminergic signals and associated with Parkinson's disease [76], and in the hippocampus [64, 77]. At the cellular level, the AT_2_ receptor is expressed in neurons, but not in astrocytes [28, 72, 78]. Evidence also suggests that the AT_2_ receptor is expressed in the vasculature wall, where it acts on cerebral blood flow (review in [31, 49]). In addition, the presence of a non-AT_1_/non-AT_2_ receptor in the CNS has been suggested, which displays high affinity for Ang I, II, and III [79].

Interestingly, the Ang IV receptor IRAP is also observed in structures classically associated with cognitive processes and sensory and motor functions (including hippocampus, thalamic nuclei, caudate putamen, cerebellum, neocortex, lateral geniculate body, inferior olivary nucleus, superior colliculus, ventral tegmental area, and brain stem) (review in [12, 80, 81]). In contrast, the Ang (1–7) Mas receptor is expressed in brain areas involved in central cardiovascular regulation (in particular, the nucleus of the solitary tract (NTS), rostral ventrolateral medulla (RVLM), caudal ventrolateral medulla (CVLM), inferior olive, parvo- and magnocellular portions of the paraventricular nucleus (PVN), supraoptic nucleus, and lateral preoptic area) (review in [82]). In some instances, these areas also contain AT_1_ and AT_2_ receptors.

One of the first roles of the AT_2_ receptor to be identified was the modulation of neuronal excitability (review in [28, 83]). In cells of neuronal origin, activation of the AT_2_ receptor decreases activity of T-type calcium channels [84, 85] while stimulating a delayed-rectifier K^+^ current (*I*_*K*_) and a transient K^+^ current (*I*_*A*_) [86]. Using primary cultures of cortical neurons, studies by Grammatopoulos et al. [87] have shown that II neuroprotection against chemical hypoxia was mediated by activation of a delayed rectifier K^+^ channel, an effect exemplified by simultaneous blockade of the AT_1_ receptor. Moreover, in preparations of locus coeruleus brain slices [88], angiotensin II, through the AT_2_ receptor, was found to depress glutamate depolarization and excitatory postsynaptic potentials. In the superior colliculus, both AT_1_ and AT_2_ receptors are involved in sensory visuomotor integration. Lastly, Ang II, through AT_2_ receptor stimulation with CGP42112A, has also been shown to induce a strong suppressive effect on visual neuronal activity. Together, these results indicate that AT_2_ receptor modulation of potassium and calcium channels activity may impact neuronal functions.

**Figure 3:**
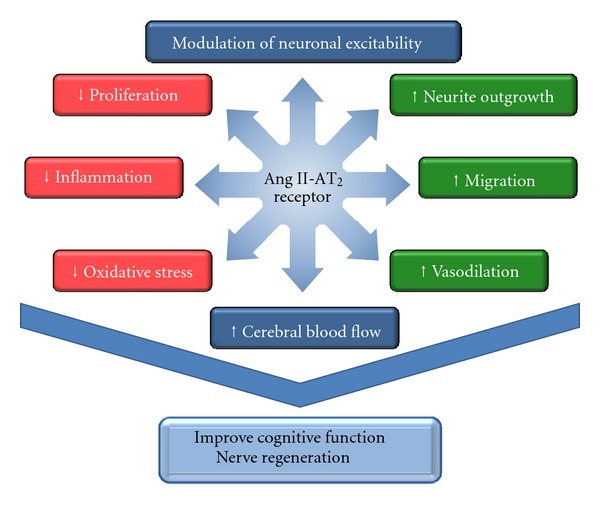
Summary of the known cellular effects of the AT_2_ receptor. Stimulation of the AT_2_ receptor of angiotensin II results in the activation of several intracellular cascades, resulting in a decrease in proliferation and in growth-promoting effects, as well as a decrease in inflammatory cytokines and in reactive oxygen species, hence decreasing oxidative stress. On the other hand, such activation increases neuronal excitability, by acting on K^+^ and Ca^2+^ channel activity, and increases neurite outgrowth and migration of neurons, favoring neuronal plasticity. Stimulation of the AT_2_ receptor is also known for its effect on vasodilation, thus increasing cerebral blood flow, and to increase glucose uptake, which improves insulin sensitivity. These cellular events in turn decrease or increase important physiological functions, which in general have reached a significant degree of consensus. Together, the AT_2_ receptor may appear as a gatekeeper of cellular and tissue homeostasis. Indeed, most current studies suggest that AT_2_ receptor activation may have a protective role in various pathological situations.

The second and best recognized effect of the AT_2_ receptor is stimulation of neurite outgrowth in various cell types from neuronal origin (NG108-15 and PC12W cells) as well as in primary cultures of neurons from retinal explants [89], in neurospheres from mouse fetal brain [90], cerebellar neuronal cells [91], and the cortex [47] (review in [3, 10, 14, 34]). This AT_2_-induced neurite elongation is characterized by an increase in mature neural cell markers, such as *β*III-tubulin and MAP2 [47, 92]. It is also associated with a rise in neuronal migration [54, 91] and neuronal survival following ischemia-induced neuronal injury [93]. These effects may be important not only for developmental differentiation, but also after injury-induced regeneration. Indeed, a beneficial effect of the AT_2_ receptor in nerve regeneration has been observed following both optic [89] and sciatic [94] nerve crush or in perivascular nerves implicated in vasodilation [95]. This implication of the AT_2_ receptor in neuronal regeneration has even led to the suggestion that Ang II, via the AT_2_ receptor, could act as a neurotrophic factor. In summary, the AT_2_ receptor, by its function in the modulation of neuronal excitability, neurite elongation, migration, and nerve regeneration, may be an important factor in the regulation of central nervous system activity and cognitive function either following nerve injury or during the development of neurodegenerative disease (Figure 3). 

In addition to neuronal differentiation, which is of paramount importance in nerve regeneration, the AT_2_ receptor also stimulates differentiation of hematopoietic cells, a key process during regeneration and reconstruction. Indeed, ischemic damage is characterized by infiltration of a number of hematopoietic cells such as leukocytes, platelets, macrophages, and leukocytes [96]. In particular, the AT_2_ receptor has the capacity to induce differentiation of human monocytes into dendritic cells [97]. Supporting a protective effect of the AT_2_ receptor is the observation that ischemic damages were found to be greater in mice with hematopoietic cells deleted in AT_2_ receptor expression [98]. These findings suggest that expression and activation of the AT_2_ receptor in hematopoietic cells may be part of its beneficial effect following brain injury, although the mechanism involved remains to be investigated (review in [99]).

Some of the initial hypotheses regarding the potential role of the AT_2_ receptor *in vivo* were confirmed in 1995 when two independent groups developed AT_2_ knockout mice by targeted gene deletion [100, 101]. Surprisingly, despite the fact that the AT_2_ receptor is highly expressed during fetal development in many tissues, including skin, kidney, brain, and heart, it appears that mice lacking the AT_2_ receptor do not exhibit any major anatomical defects. However, these mice exhibit markedly reduced exploratory behavior as well as altered thirst reaction, lower body temperature, slightly elevated mean arterial pressure, and a stronger vasoconstrictive response to Ang II [100–102]. Moreover, they exacerbate stronger symptoms when exposed to pathological situations. For example, these mice display an accelerated pathological response when exposed to cardiovascular disease induction. They also exhibit larger cerebral infarct size following medial cerebral artery occlusion (MCAO) [93], stronger cognitive deficits following ischemia [103], and faster progression of atherosclerosis [104]. The various actions of the AT_2_ receptor currently documented in the brain are summarized in Figure 3. 

## 5. Role of the AT_2_ Receptor in Cognitive Function and Neurological Disorders 

The first evidence for the implication of the AT_2_ receptor in cognition resulted from studies in knockout mice. Indeed, AT_2_-deficient mice suffer from perturbations in exploratory behavior and locomotor activity [100, 101], as well as displaying an anxiety-like behavior [105]. Moreover, in the adult, inhibition of the AT_2_ receptor with the AT_2_ receptor antagonist PD123,319 has been reported to abolish the Ang II-induced acquisition of conditioned avoidance responses [106]. These results strongly support that, in addition to a role during development, the AT_2_ receptor may be involved in cognitive processes in the adult. Furthermore, although the AT_2_ receptor is expressed at low levels in many areas of the nervous system, it may be reexpressed in certain pathological conditions such as optic [89] or sciatic [94] nerve transection, stroke [107], and certain neurodegenerative diseases such as Alzheimer's disease [108]. For example, Ge and Barnes [108] found that AT_2_ receptor expression is diminished in Parkinson's disease (caudate nucleus and cerebellum) but enhanced in Huntington's disease (caudate nucleus). In Alzheimer's disease, the temporal cortex of the adult brain exhibits an increased expression while the hippocampus displays a decreased expression of the AT_2_ receptor. In most of these situations, the AT_2_ receptor is described as having beneficial effects in improving neuroprotection by acting not only on neurons, but also on blood circulation. Indeed, recent studies have clearly shown a protective role of the AT_2_ receptor following brain ischemia and demonstrated that expression and activation of the AT_2_ receptor may decrease brain damage and restore cognitive loss following middle cerebral artery occlusion (for review, see [11, 14, 49]). Altogether, these data suggest that the AT_2_ receptor may play an important role in maintaining functions of the human brain.

### 5.1. AGTR2 Mutations in Intellectual Disability

Intellectual disability, previously described as mental retardation [109], affects approximately 1–3% of the population, of which a large number are associated with mutations on chromosome X. Among these, certain mutations on *AGTR2* coding for the angiotensin AT_2_ receptor have been identified. Mutations in the *AGTR2 *gene correlate with the development of human X-linked intellectual disability [110]. Indeed, 9 patients with X-linked intellectual disability were shown to have mutations in the *AGTR2 *gene associated with decreased expression of the AT_2_ receptor, including a complete loss of expression in a woman with an IQ of 44. Clinical features of these mutations ranged from moderate to severe intellectual disability, seizure, and manifestations of autism, thus supporting the hypothesis that the AT_2_ receptor is required for brain development and for the maintenance of neuronal connections involved in learning and memory. This hypothesis was further corroborated by two other studies reporting mutations of *AGTR2* in patients suffering from intellectual disability, seizures, restlessness, hyperactivity, and disrupted speech development [111, 112]. However, other studies failed to link any *AGTR2* mutations with intellectual disability, observing no difference in mutation incidence between the latter and control groups [113–115]. Differences among control cohort selection, number of patients per group, and ethnical variations between different studies could explain the discrepancies in these findings. Hence, it remains unclear whether mutations in *AGTR2* are associated or not with intellectual disability.

### 5.2. AT_2_ Receptor in Alzheimer's Disease (AD)

Amyloid-*β* (A*β*) deposition in senile plaques and the presence of neurofibrillary tangles are the main pathological hallmarks of AD. However, other structural and functional alterations, including inflammation, increased oxidative stress and vascular damage/ischemia, are also associated with AD and other neurodegenerative diseases. These alterations may contribute to neuronal and synaptic dysfunction and loss, as well as the ensuing cognitive deficits and dementia of this disorder [116–121] (for recent review, see [11, 14, 49]). Clinical studies have documented that treatment with antihypertensive drugs is associated with an improvement in cognitive function (review in [122–124]). More recent studies have shown that treatment with angiotensin II receptor blockers (ARBs) is associated with a decrease in AD and dementia progression with a greater efficacy compared to ACE inhibitors [125, 126]. In particular, Tsukada et al. [127] have shown that cognitive deficit induced by A*β* (1–42) in mice was improved by pretreatment with a low dose of telmisartan partly because of peroxisome proliferator-activated receptor-gamma (PPAR*γ*) activation. Corroborating the hypothesis that ARBs could be beneficial in reducing the onset of AD, Kume et al. [128] recently observed that AD hypertensive patients treated with telmisartan presented no decrease in cognitive functions test scores, but an increased cerebral blood flow, suggesting that treatment with this ARB could reduce AD progression. Altogether, studies conducted using various models of cognitive disorders have reported improved memory and cognitive processes and/or attenuation of A*β*1–42 oligomerization following treatment with ARBs, particularly valsartan [129], losartan [130], telmisartan [128, 131], and olmesartan [132] (now called metabosartans for ARBs with a PPAR*γ* agonistic effect) (review in [11, 14, 49, 133]). More recently, a study using direct stimulation of the AT_2_ receptor with the selective agonist C21/M024 demonstrated similar effects in an AD mouse model [134]. In this latter study, Jing et al. observed that intracerebroventricular injections of A*β* (1–40) in mice induced a poorer performance in the Morris water maze and that this effect was reversed by coadministration of C21/M024, indicating that direct stimulation of AT_2_ receptors improves spatial memory functions. Stroke is also one of the most important causes of cognitive impairment and dementia (review in [11, 49]). There is increasing evidence suggesting that activation of the AT_2_ receptor could improve cerebral blood flow and microcirculation as well as decrease inflammation [93, 103, 135, 136], both being associated with improvement in cognitive function following cerebral ischemia [90, 93, 103, 137] (for reviews, see [3, 10, 14, 49]). As reported by Iwai et al. [103] and summarized by Horiuchi and Mogi [49] and Mogi and Horiuchi [11], superoxide anion production was found to be more markedly enhanced in AT_2_ receptor-deficient mice compared to wild-type animals in a model of MCAO. These observations suggest that AT_2_ receptor stimulation has a protective effect on ischemic brain lesions, at least partly through modulation of cerebral blood flow and superoxide production. Moreover, beneficial effects of ARBs on these parameters were less evident in AT_2_ receptor-deficient mice [103]. Similar approaches were also used in other studies suggesting a beneficial effect of the AT_2_ receptor on cognitive functions [90, 135, 136]. 

However, not only blood circulation but also neuronal functions can be improved by activation of AT_2_ receptors. Indeed, activation of AT_2_ receptors in neurons is also associated with a decrease in apoptosis signaling. Grammatopoulos et al. demonstrated in cultures of primary cortical neurons that angiotensin decreased sodium azide-induced apoptosis through AT_2_ receptor activation [138] by reducing caspase-3 activation [139]. Finally, we and others have shown that the AT_2_ receptor can activate the tyrosine kinase Fyn [44] and the phosphatase PP2A [51, 52, 54], both of which are key regulators of the phosphorylation of the microtubule associated protein tau. Hyperphosphorylation of tau is of paramount importance in the development of Alzheimer's disease by forming neurofibrillary tangles and leading to microtubule depolymerization. Thus, AT_2_ receptor activation may participate to the control of equilibrium between tau phosphorylation and dephosphorylation. 

Another key observation is the effect of estrogen receptors on AT_2_ receptor functions. In 2008, Chakrabarty et al. [140] demonstrated that estrogen, through its E2 receptor, induced neurite elongation of dorsal root ganglion and that this effect was dependent on AT_2_ receptor activation. Moreover, it has been observed that ischemic damage in AT_2_-deficient mice was greater in females than in males, while no significant sex-different change was observed in AT_2_-expressing mice [135]. These results suggest that the existence of some level of crosstalk between AT_2_ receptor and estrogen and that AT_2_ receptor could be necessary for E2 receptors to elicit its full effect on neuronal physiology. Thus, while stimulation of the AT_2_ receptor and/or inhibition of the AT_1_ receptor could lead to potential therapeutic avenues in neurodegenerative disease, the interaction between AT_2_ receptor and estrogen should also be considered. Indeed, there are some differences in response to RAS stimulation according to gender (review in [141]). Nonetheless, the clear demonstration of an AT_2_ receptor effect in Alzheimer's disease remains to be firmly demonstrated, mainly because in most studies to date the presence of AT_1_ receptors and AT_2_ receptors in the hippocampus has not been studied. Indeed, to our knowledge, only two studies have documented the presence of AT_2_ receptors in the hippocampus, one in a model of Alzheimer's disease [64], the other in a model of epilepsy [77]. On the other hand, presence of the AT_2_ receptor was not detected in the hippocampus in various studies from the Llorens-Cortes group [72, 73]. However, these studies were performed with healthy and middle-aged animals. 

It should be mentioned that, in addition to the AT_2_ receptor, the Ang IV/AT_4_ receptor may also have a protective effect on cognitive function. Indeed, Braszko's group was the first to report that intracerebroventricular injections of Ang II and Ang IV were equivalent in facilitating exploratory behavior in rats tested in an open field and improved recall of passive avoidance conditioning in the acquisition of active avoidance conditioning [142, 143], results that were confirmed by others in subsequent studies (review in [71]). This strongly suggests an important function of Ang IV and its receptor in learning and memory processes and could represent a new therapeutic target in the treatment of memory loss associated with dementia (review in [71]). One mechanism proposed to explain this beneficial effect of Ang IV on cognitive function is the colocalization of IRAP with the glucose transporter GLUT4. In hippocampal pyramidal neurons, IRAP and GLUT4 are localized in secretory vesicles responsive to insulin (review in [70, 71, 80]), suggesting that Ang IV, by binding to IRAP, may increase GLUT4 membrane expression thus facilitating glucose uptake, as observed in adipocytes [144] (review in [145]).

### 5.3. AT_2_ Receptor and Parkinson's Disease

In addition to AD and stroke, some evidences also suggest that central RAS could be implicated in the development of Parkinson's disease. Parkinson's is the second most common neurodegenerative disorder and is characterized by the progressive cell death of midbrain dopaminergic neurons in the substantia nigra and the presence of protein inclusions leading to formation of Lewy bodies (review in [146]). Although the mechanisms leading to Parkinson's disease are still unclear, it appears that mitochondrial dysfunction, oxidative stress, and inflammation are key factors to its progression [147]. Recently, ARBs have been shown to reduce lipid peroxidation and protein oxidation while protecting dopaminergic neurons in the substantia nigra in a rat model of Parkinson's disease [76, 148]. However, whether such action is due solely to a blockade of the AT_1_ receptor or also from activation of the AT_2_ receptor is not yet clearly established (review in [147]). Activation of the AT_2_ receptor is able to stimulate differentiation of mesencephalic precursor cells into dopaminergic neurons, suggesting that stimulation of the AT_2_ receptor could be useful in increasing the production of dopaminergic neurons in Parkinson's disease [149]. Moreover, Grammatopoulos et al. observed a protection against rotenone-induced oxidative stress and associated cell death in dopaminergic neurons following Ang II stimulation. This protective effect was prevented by the presence of the AT_2_ receptor antagonist PD123,319, but was increased in the presence of the AT_1_ receptor antagonist losartan [150]. More recently, it has been observed that during the aging process in rats, AT_2_ receptor expression is decreased in dopaminergic neurons, as opposed to an increase in AT_1_ receptor expression. This was associated with an enhancement of prooxidative and proinflammatory markers in the substantia nigra, leading to an increase in dopaminergic neuronal death [151]. Although these findings do not indicate a role of AT_2_ receptor in the development of Parkinson's disease, they strongly suggest that such modifications in Ang II receptors during the natural aging process may increase the risk of Parkinson's disease.

## 6. Role of the AT_2_ Receptor in the Regulation of Appetite

Obesity, which is characterized by excess body fat accumulation [152], is associated with an increased risk of diabetes, hypertension, and dyslipidemia. It is also one of the major components of the metabolic syndrome. Studies have demonstrated AT_2_ receptor expression in tissues associated with glucose metabolism, including pancreatic [153–155] and adipose tissues [13, 30]. For example, expression of the AT_2_ receptor in the pancreas has been shown to be important for fetal pancreatic development [156] while in the adult, it is associated with protection against pancreatic fibrosis [157] and a decrease in pancreatic tumor growth [158, 159]. The following is a summary of what is currently known regarding the potential function of the AT_2_ receptor in the regulation of appetite, glucose metabolism, and its potential role in metabolic syndrome.

There are some evidences suggesting that Ang II could be implicated in food intake: for example, Ang II suppresses food intake after central infusion [32, 160, 161] while blockade of the AT_1_ receptor by telmisartan is associated with a decrease in body weight [162]. Furthermore, both AT_1_ and AT_2_ receptors are expressed in the hypothalamus, which is implicated in the central regulation of food intake. In 2008, Ohinata et al. [32] observed that the decrease in food intake induced by centrally administrated Ang II was inhibited by PD123,319, and absent in AT_2_-KO mice, suggesting that this effect was mediated by the AT_2_ receptor. A similar effect was observed using novokin, a potent analog of ovokinin with AT_2_ receptor agonistic properties [163]. Although the mechanism underlying this AT_2_ receptor-associated decrease in food intake remains unclear, it may be linked to its capacity to modulate T-type calcium channels, since a recent study demonstrated that inhibition of these channels inhibits weight gain in mice fed with a high-fat diet [164]. However, this hypothesis remains to be explored. Studies conducted to date with the selective AT_2_ agonist C21/M024 do not describe such differences between C21/M024-treated animals compared to the control group [165, 166], suggesting that the duration of C21/M024 treatment may have been too short to induce any modification in body weight. Thus, in this latter instance, even if the AT_2_ receptor was observed to decrease food intake, it was probably not sufficient to induce a decrease in body weight, at least following short periods of stimulation (<8 weeks). Moreover, it has also been observed that inhibition of ACE by captopril also reduced body weight of mice fed with a high fat diet [167]. This decrease in body weight, however, was not associated with a decrease in food intake, suggesting that other mechanisms were regulated by Ang II. Since Ang II is no longer available in captopril-treated mice, these results suggest that other members of the RAS, independent of Ang II, could be implicated in the regulation of food intake and body weight. These observations should therefore be considered when interpreting results obtained with ARBs.

## 7. Link between Metabolic Syndrome and Alzheimer's Disease: Is There a Place for the AT_2_ Receptor?

A number of excellent reviews have recently been published regarding the potential link between insulin resistance, metabolic syndrome, and neurodegenerative disorders (both AD and vascular dementia) [168–177], including the involvement of RAS in this process [90, 133, 178]. Development of AD is closely associated with a reduction in cerebral glucose utilization, even in the early stages of the disease. In fact, cerebral metabolism in the AD brain decreases prior to the onset of cognitive decline, suggesting that energy failure could represent one of the earliest hallmarks of AD. Induction of insulin resistance in AD animal models aggravate both amyloid and tau accumulation [179, 180], leading several investigators to refer to Alzheimer's disease as type 3 diabetes (review in [168]). One aspect of this relationship is the loss of insulin signaling in the insulin-resistant brain. In addition to the many peripheral complications associated with dysfunction in insulin sensitivity, it appears that brain insulin signaling plays crucial central functions in the regulation of energy balance (food intake, body weight) as well as in learning and memory (review in [171]). Moreover, inflammation, increase in oxidative stress, and mitochondrial dysfunctions are key features of type 2 diabetes (T2D) that are also shared in Alzheimer's disease. 

In T2D patients, results of a major clinical study (Study on Cognition and Prognosis in the Elderly, SCOPE) [181] and a clinical double-blind study [182] have revealed that ARBs have a further therapeutic effect on impaired cognitive function beyond their antihypertensive effects compared with other antihypertensive drugs. Similarly, Tsukuda et al. [131, 183] have demonstrated that candesartan improves impaired cognitive function induced by T2D, with multiple beneficial effects. Two of these effects may be through PPAR*γ* or through AT_2_ receptor activation. Therefore, improvement of metabolic syndrome may also be beneficial in decreasing associated cognitive decline. This would contribute to better insulin signaling in the brain and, therefore, a slowing of cognitive decline associated with brain insulin resistance.

## 8. New Insights in AT2 Receptor Knowledge and Perspectives: What Remains to Be Done? 

As pointed out recently [18, 20], there is still an ongoing debate as to the putative role of the AT_2_ receptor in physiology, and whether this role is deleterious or beneficial. Summarized below are some of the new advances in AT_2_ receptor signaling that could have important insights in AT_2_ receptor-associated brain functions. 

### 8.1. Homo- and Heterodimerization

Although GPCRs have traditionally been thought to act as monomers (review in [184]), it is now well accepted that many GPCRs can form dimers which could affect both their trafficking and function. In this context, homodimerization of AT_1_ and AT_2_ receptors, as well as AT_1_/AT_2_ heterodimer formations, has been reported. For example, the AT_2_ receptor is known to undergo homodimerization, a property which enhances apoptosis [185]. In addition, AbdAlla et al. [186] reported that the AT_2_ receptor undergoes heterodimerization with the AT_1_ receptor in transfected PC12 cells, in fetal fibroblasts, and in myometrial biopsies. In an animal model of Alzheimer's disease, the same group demonstrated that A*β* induces the formation of cross-linked AT_2_ receptor oligomers [64, 187]. Notably, oligomers of AT_2_ receptors have also been observed in prefrontal cortex specimens of Alzheimer's disease patients, while being completely absent in specimens of nondemented control individuals, thus lending further support for a role of the AT_2_ receptor in cognitive function. Heterodimerization between the AT_2_ receptor and bradykinin has also been described in PC12W cells [188]. It is already known that bradykinin mediates AT_2_ receptor-induced NO production [189–191]. This interaction between the two receptors has been shown to enhance phosphorylation of various kinases, including p42/p44^mapk^ and p38^mapk^. Therefore, it would appear that homo- and heterodimerization of the AT_2_ receptor may have a role in its regulation. However, these initial observations still require confirmation before being accepted as important regulatory aspects of Ang II receptor signaling and functions (review in [29, 31]). The use of recently developed methodologies such as FRET/BRET technology has confirmed efficient heterodimerization of the AT_1_ receptor with the bradykinin receptor B2 [192], indicating the potential clinical significance of GPCR oligomerization [29, 31]. Moreover, recent studies have identified intracellular crosstalk pathways between the AT_1_ receptor and the AT_2_ receptor at the gene expression level. Indeed, AT_1_ receptor activation enhances AT_2_ receptor mRNA degradation, while AT_2_ receptor activation increases its own mRNA transcription [193].

### 8.2. PPAR*γ*: Could It Be the Missing Link?

There is existing confusion regarding the mechanism of action of specific ARBs, since some also have partial PPAR*γ* agonistic activity (such as telmisartan, irbesartan, and candesartan). There is some evidence suggesting that this PPAR*γ* activation following blockade of the AT_1_ receptor could be part of its anti-inflammatory and antioxidative effects, leading to neuroprotection against ischemia and A*β* accumulation [127, 194, 195]. Indeed, neuroprotective effects of PPAR*γ* agonists, such as pioglitazone, have been observed during neural cell differentiation and death, and in inflammatory and neurodegenerative conditions, including amyotrophic lateral sclerosis, Alzheimer's disease and Parkinson's disease models, as well as stroke [196, 197]. PPAR*γ* is a transcriptional factor regulating the expression of multiple genes, thereby promoting the differentiation and development of various tissues, specifically adipose tissue, brain, placenta, and skin (review in [197]). In addition, certain studies have indicated that AT_2_ receptor stimulation increases PPAR*γ* expression and transcriptional activity, at least in PC2W cells [57] and neurons [131]. The final targets of these pathways are gene expression and phosphorylation of various microtubule-associated proteins, which modulate microtubule stability/dynamics responsible for neurite elongation. This observation is noteworthy, especially with regard to the implication of PPAR*γ* in NGF-induced neurite outgrowth [198] which clearly suggests a possible crosstalk between the AT_2_ receptor and NGF pathways. This hypothesis is furthermore reinforced by the observation that inhibition of the NGF receptor TrkA significantly decreases AT_2_ receptor-induced neurite outgrowth [43]. Moreover, Iwai et al., using atherosclerotic ApoE-KO mice with an AT_2_ receptor deficiency (AT2R/ApoE double knockout mice), observed that the lack of AT_2_ receptor expression decreased the expression of PPAR*γ* in adipocytes [199]. These observations strongly suggest a link between the AT_2_ receptor and PPAR*γ* functions. Considering that similar neuroprotective effects were also associated with the AT_2_ receptor, it may be hypothesized that activation of PPAR*γ* could be shared both by ARBs and AT_2_ receptor stimulation. 

## 9. Could the AT_2_ Receptor Be an Attractive Therapeutic Target?

One of the biggest challenges in studying the AT_2_ receptor is applying observations stemming from the use of cell lines to *in vivo* models. Indeed, studies using cell lines expressing the AT_2_ receptor either endogenously or via transfection have provided paramount information regarding its intracellular mechanisms of action. However, associating these mechanisms with biological functions has proven to be much more difficult. As indicated previously, synthesis and characterization of the selective AT_2_ receptor selective agonist C21/M024 in 2004 or of the recently developed antagonist [200] has provided long-awaited tools to bypass the difficulty of using traditional AT_2_ receptor ligands such as CGP42112A or PD123,319. Since then, many studies have allowed significant advances in the understanding of AT_2_ receptor functions. Nonetheless, one would have thought that this new AT_2_ receptor ligand would have resolved certain controversies surrounding this enigmatic receptor. However, eight years after the first characterization of this compound, the clear demonstration of an AT_2_ receptor effect in the brain remains to be unequivocally established. One major input since C21/M024 was first described is that selective stimulation of the AT_2_ receptor does not decrease blood pressure [134, 165, 166, 201–206]. These results are quite surprising, considering previous reports emanating from the indirect *in vivo* manipulation studies which associated AT_2_ receptor activation with vasodilation and a decrease in mean arterial pressure. However, blockade of the AT_1_ receptor with ARBs not only allows stimulation of the AT_2_ receptor, the only Ang II receptor available in this condition, but also increases the bioavailability of Ang II for ACE2 and aminopeptidase to produce Ang IV and Ang (1–7), both of which exert vasodilatory effects (Figure 1). Nevertheless, beyond its blood-pressure lowering effects, *in vivo* studies using C21/M024 have described a protective role of the AT_2_ receptor in vascular remodeling [166, 207], in poststroke cardiac [208] and renal function [165, 205] as well as in cognitive functions [134]. Furthermore, observation of AT_2_ receptors in tissues associated with glucose metabolism, such as the pancreas and adipose tissue, suggests that the AT_2_ receptor could also be beneficial in metabolic syndrome and associated cognitive loss. However, the selectivity of C21/M024 for the AT_2_ receptor has also been recently challenged and differs according to the dosage used and/or route of administration and, importantly, according to experimental conditions. In particular, a recent observation by Verdonk et al. [209], whereby C21/M024 induced vasorelaxation of preconstricted iliac arteries from SHR Wistar rats and C57BL/6 mice as well as in AT_2_-deficient mice, has rekindled the debate on the relevance of the AT_2_ receptor and the selectivity of AT_2_ receptor ligands in physiological functions. Moreover, this effect of C21/M024 in arteries was only partially blocked by PD123,319. These latest findings raise the possibility that C21/M024 could exhibit certain AT_2_ receptor-independent effects. Therefore, the question still remains: is the AT_2_ receptor a potential therapeutic target and could it replace or increase beneficial effects associated with ARBs? 

## 10. Conclusion

As described in the aforementioned sections, AT_2_ receptor activation may act at several stages in the cascade of alterations leading to cognitive impairment and neuronal dysfunction. An increasing number of studies suggest that the protective effects of ARBs on brain damage and cognition may result not only from the inhibition of AT_1_ receptor effects, but also from the beneficial effect due to unopposed activation of the AT_2_ receptor. In addition, the relationship between impaired energy metabolism/obesity/insulin resistance and the increased risk of dementia emphasizes the view that the mechanisms of action of the AT_2_ receptor may have a beneficial protective effect. However, the physiological relevance of the AT_2_ receptor in the brain will need to be compared with at least two other components of RAS, namely, the ACE2/Ang-(1–7)/Mas complex and Ang IV/AT_4_ receptor/IRAP in the brain (Figure 1).

Lifestyle-related disorders, such as hypertension, T2D, and obesity, are also implicated as risk factors for dementia. In this regard, two recent publications have established that direct AT_2_ receptor stimulation with C21/M024 improves insulin sensitivity in a rat model of diet-induced insulin resistance [210] and in type 2 diabetic mice [211]. Thus, any treatment aimed at improving insulin resistance or cognitive functions is likely to slow down symptoms and improve quality of life associated with these age-related disorders. Furthermore, the recent development of selective AT_2_ receptor agonists should facilitate efforts to elucidate distinct roles of the AT_2_ receptor in brain physiology, supporting or disproving the hypothesis that the AT_2_ receptor helps improve a number of brain impairments related to neuronal plasticity and morphology, microcirculation and inflammation (Figure 3), all of which are altered in certain neurological disorders.

There is clearly still much work to be accomplished to fully understand the role of the AT_2_ receptor in normal versuspathological conditions and to determine whether AT_2_ receptor agonists could represent an attractive therapeutic target. In this aspect, compounds such as C21/M024 as well as other recently synthesized highly selective nonpeptide AT_2_ receptor ligands (all leading to neurite outgrowth in NG108-15 cells) and their effectiveness to induce AT_2_ receptor-dependent effects need to be further explored [212–216].
